# Biochemical analysis and the preliminary crystallographic characterization of d-tagatose 3-epimerase from *Rhodobacter sphaeroides*

**DOI:** 10.1186/s12934-017-0808-4

**Published:** 2017-11-09

**Authors:** Zhengliang Qi, Zhangliang Zhu, Jian-Wen Wang, Songtao Li, Qianqian Guo, Panpan Xu, Fuping Lu, Hui-Min Qin

**Affiliations:** 1 Key Laboratory of Industrial Fermentation Microbiology, Ministry of Education, Tianjin, People’s Republic of China; 2 Tianjin Key Laboratory of Industrial Microbiology, Tianjin, People’s Republic of China; 3 College of Biotechnology, Tianjin University of Science and Technology, Tianjin, People’s Republic of China; 4 National Engineering Laboratory for Industrial Enzymes, Tianjin, 300457 People’s Republic of China

**Keywords:** d-Tagatose 3-epimerase, d-Fructose, d-Psicose, Crystallization, Structural analysis, TIM-barrel fold

## Abstract

**Background:**

d-Tagatose 3-epimerase epimerizes d-fructose to yield d-psicose, which is a rare sugar that exists in small quantities in nature and is difficult to synthesize chemically. We aim to explore potential industrial biocatalysts for commercial-scale manufacture of this rare sugar. A d-tagatose 3-epimerase from *Rhodobacter sphaeroides* (RsDTE) has recently been identified as a d-tagatose 3-epimerase that can epimerize d-fructose to yield d-psicose with a high conversion rate.

**Results:**

The purified RsDTE by Ni-affinity chromatography, ionic exchange chromatography and gel filtration forms a tetramer in solution. The maximal activity was in Tris–HCl buffer pH 8.5, and the optimal temperature was at 35 °C. The product, d-psicose, was confirmed using HPLC and NMR. Crystals of RsDTE were obtained using crystal kits and further refined under crystallization conditions such as 10% PEG 8000,0.1 M HEPES pH 7.5, and 8% ethylene glycol at 20 °C using the sitting-drop vapor diffusion method. The RsDTE homology model showed that it possessed the characteristic TIM-barrel fold. Four residues, Glu156, Asp189, Gln215 and Glu250, forms a hydrogen bond network with the active Mn(II) for the hydride transfer reaction. These residues may constitute the catalytic tetrad of RsDTE. The residues around O1, O2 and O3 of the substrates were conserved. However, the binding-site residues are different at O4, O5 and O6. Arg118 formed the unique hydrogen bond with O4 of d-fructose which indicates RsDTE’s preference of d-fructose more than any other family enzymes.

**Conclusions:**

RsDTE possesses a different metal-binding site. Arg118, forming unique hydrogen bond with O4 of d-fructose, regulates the substrate recognition. The research on d-tagatose 3-epimerase or d-psicose 3-epimerase enzymes attracts enormous commercial interest and would be widely used for rare sugar production in the future.

**Electronic supplementary material:**

The online version of this article (10.1186/s12934-017-0808-4) contains supplementary material, which is available to authorized users.

## Background

d-Psicose, a rare sugar that exists in small quantities in nature, is a valuable low-calorie sweetener food additive [1, 2] due to its poor absorbance in the digestive tract and almost no energy. The supplemental d-psicose might be helpful for diabetic patients in preventing post prandial hyperglycemia [3, 4]. d-Psicose is commercially produced by alkali isomerization. However, biosynthesis of rare sugars is environmentally friendly and sustainable with moderate reaction conditions and high efficiency compared with chemical synthesis [5, 6]. Therefore, the most promising approach to produce d-psicose is to use an enzymatic reaction catalyzed by d-psicose-tagatose 3-epimerase (DTE) or d-psicose 3-epimerase (DPE) [6–8]. DTE and DPE were named based on the substrate specificities toward d-tagatose or d-psicose, respectively. Both of these enzymes catalyze the epimerization of various ketohexoses, such as d-tagatose and d-fructose to d-sorbose and d-psicose, respectively, by catalyzing the epimerization at carbon-3 (C3) position [9–11].

The characterization study of DTE/DPE from *Pseudomonas cichorii* (PcDTE, 290 amino acid residues, 32,615 Da) and *Agrobacterium tumefaciens* (AtDPE, 283 amino acid residues, 30,650 Da) was reported to efficiently catalyze epimerization of not only d-tagatose to d-sorbose, but also d-fructose to d-psicose [12, 13]. AtDPE has a sequence similarity of 39% with PcDTE. However, there are significant differences in enzymatic properties between them. For instance, PcDTE shows the highest epimerization activity toward d-tagatose, while AtDPE has higher bioconversion rate toward d-psicose than PcDTE [13]. Furthermore, the crystal structures of AtDPE and PcDTE were reported [14, 15], suggesting that both enzymes showed a (β/α)_8_ TIM barrel fold with a Mn^2+^ metal ion in the active site. Two glutamate residues and a metal ion conduct the epimerization reaction analogous to the catalytic mechanism of d-ribose-5-phosphate 3-epimerase. Protein engineering was performed on PcDTE to improve its catalytic activity using its structural information [16]. Based on the structural information [8, 14, 15, 17, 18], DTE and DPE shared the same catalytic mechanism of deprotonation/protonation at C3 by two Glu residues. One of the Glu residues removes a proton from C3 to generate a cis-enediolate intermediate, and then the other one protonates C3 on the opposite side.

A novel identified DTE from *Rhodobacter sphaeroides* (RsDTE) has shown the highest activity for d-fructose with a bioconversion rate to d-psicose of 23% at 40 °C and pH 9.0 [11]. However, the reason why RsDTE showed a high specificity for d-fructose remains unclear. The structure analysis will provide more information for the catalytic reaction. In this study, the RsDTE was purified, crystallized and characterized. The substrate-binding site was analyzed based on RsDTE homology model. The research on DTE/DPE enzymes attracts enormous commercial interest and would be widely used for rare sugar production in the future.

## Methods

### Cloning and expression

Genomic DNA of DTE was prepared from *R. sphaeroides* strain as previously described [11] and was used as a template for genomic polymerase chain reaction (PCR) with the PrimeSTAR HS DNA polymerase (TaKaRa, Dalian, China) and a pair of specific primers, F: (5′-GGAATTCCATATGAAAAATCCTGTCGGCATCATCTCG-3′) and R: (5′-CCGGAATTCTCAGTGGGTCACCTCCGCC-3′), for the 5′- and 3′-untranslated regions, respectively. PCR was conducted using temperature settings of 95 °C for 5 min followed by 30 cycles of 95 °C for 15 s, 55 °C for 15 s and 72 °C for 45 s. The final step for extension was 72 °C for 2 min. The RsDTE gene (GenBank Accession No. NC_007494.2) was cloned into the vector pET28a(+) (Novagen, Madison, WI, USA) between the *Nde*I and *Eco*RI sites (Additional file 1: Figure S1) with 20 residues (MGSSHHHHHHSSGLVPRGSH) at the N-terminus, including His_6_ tag and thrombin digestion site. *E. coli* BL21(DE3) cells harboring the pET28a(+) plasmid with the RsDTE gene (pET28a-RsDTE) were transformed and grown in Lysogeny broth (LB) at 37 °C. Isopropyl β-d-1-thiogalactopyranoside (IPTG) was added at a final concentration of 0.5 mM when the OD_600_ value reached 0.6, and the cultures were further incubated at 25 °C overnight.

### Purification of RsDTE enzyme

After harvesting by centrifugation at 5000*g* and 4 °C for 15 min, the cells were resuspended in 100 mL lysis buffer (20 mM Tris–HCl pH 8.0, 10 mM imidazole, 0.5 M NaCl, and 1 mM dithiothreitol), disrupted by sonication using an ultrasonicator, set at 1 s pulse, 1 s output and 50% duty cycle for 30 min, and the cell debris was removed by centrifugation at 40,000*g* at 4 °C for 30 min. Cleared lysate was trapped on 3 mL of Ni–NTA Superflow resin (Qiagen, Hilden, Germany). After washing with 10 mL lysis buffer, the protein was eluted with 15 mL elution buffer (20 mM Tris–HCl pH 8.0, 300 mM imidazole, 100 mM NaCl, and 1 mM dithiothreitol). The eluted solution was dialyzed against 20 mM Tris–HCl pH 8.0, 1 mM dithiothreitol and was further purified by ion exchange using Source Q 4.6/100 PE (volume: 1 mL, flow rate: 3 mL/min, GE Healthcare) and gel filtration chromatography using a Superdex200 column 10/300 GL (volume: 24 mL, flow rate: 0.5 mL/min, GE Healthcare) in running buffer of 20 mM Tris–HCl pH 8.0, 100 mM NaCl, and 1 mM dithiothreitol [19, 20]. The concentration of total protein after each purification step was determined by the BCA assay following to the manufacturer’s protocol. The amount and purity of target protein were analyzed by SDS–PAGE and densitometry of CBB-stained gels using Image Lab Software (Bio-Rad, Hercules, California, USA). The resulting solution containing RsDTE was used for activity assays.

### CD measurement

Circular dichroism (CD) spectra (190–250 nm) were recorded using a MOS-450 CD spectropolarimeter (Biologic, Claix, Charente, France) with a 1 mm path-length cell at room temperature. The spectra were obtained as the average of four scans with a bandwidth of 0.1 nm, a step resolution of 0.1 nm and a scan rate of 1 nm/s. The CD spectra of RsDTE (0.08 mg/mL) were recorded in 20 mM Tris–HCl (pH 8.0), 0.1 M NaCl, and 1 mM DTT. Analysis of the protein secondary structure was performed using the program SELCON3 (http://www.dichroweb.cryst.bbk.ac) [21].

### Confirmation of the product d-psicose using HPLC and NMR

The product d-psicose was analyzed on a high-performance liquid chromatography (HPLC) system equipped with a Prevail Carbohydrate ES column-W (5 μm, 4.6 × 250 mm, Agela Technologies, China), an Agilent (USA) multichannel interface, and a XWK-III pump. Acetonitrile (85%) was used as the eluent at a flow rate of 1 mL/min. The column temperature was kept at 40 °C. The product was detected using an evaporative light-scattering detector (Agilent 1260 Infinity, USA).

The ^1^H and ^13^CNMR spectra were recorded in D_2_O on a Bruker AV-500 spectrometer at working frequencies 400 and 100 MHz, respectively. Chemical shifts are expressed in ppm (δ) values and coupling constants (*J*) in Hz.

### Activity assay

The activity was determined by measuring d-psicose formation using d-fructose as a substrate [22, 23]. The reactions were performed carried out with 5 μM RsDTE, 10 g/L d-fructose and 10 μM Mn^2+^ in 20 mM Tris–HCl buffer (pH 8.5) at 35 °C for 10 min. After the reaction, d-psicose was measured using HPLC.

The optimal pH of the purified enzyme was determined in the following buffers: 20 mM MES buffer (pH 5.5–6.5), 20 mM PBS buffer (pH 7.0–8.0), 20 mM Tris–HCl buffer (pH 8.5–9.0) and 20 mM CAPS buffer (pH 9.5–11.0) at 35 °C for 10 min. The optimal temperature was determined by incubating RsDTE at different temperatures ranging from 20 to 70 °C. To determine the effect of metal ions on the activity of RsDTE, 0.1 mM final concentrations of Ca^2+^, Mg^2+^, Mn^2+^, Ni^2+^, Co^2+^, Zn^2+^, Cu^2+^, Fe^2+^ Fe^3+^ and EDTA were added to the reaction system. The activity was then measured under standard reaction conditions. All assays were repeated three times, and the data are shown as mean ± SD.

### Crystallization of RsDTE

Crystallization of RsDTE was performed with the sparse-matrix screening kits Crystal Screen HT (Hampton Research), Index HT (Hampton Research), Wizard I and II (Emerald BioSystems) and JCSG+ (Qiagen) in 96-well plates using the sitting-drop vapour-diffusion method. For refinement of the crystallization conditions, 0.5 μL of protein was mixed with an equal volume of reservoir solution and equilibrated against 0.5 μL of reservoir solution at 20 °C in 96-well plates. The X-ray diffraction data for RsDTE crystals were collected using an in-house X-ray diffractometer (Rigaku FR-E rotating-anode X-ray generator with R-AXIS VII imaging-plate detector).

### Structure modeling of RsDTE

The three-dimensional (3D) homology model of RsDTE was generated using Modeller 9.9 [24]. The crystal structure of Pc*DTE* (PDB ID: 2QUL, 1.79 Å), which has 31% sequence identity to the target protein RsDTE, was chosen as the template. The align2d command was used to automatically generate a sequence alignment between the template and RsDTE. Subsequently, homology modeling was performed by the automodel command. Thereafter, each model was first optimized by the variable target function method with conjugate gradients. Simulated annealing MD simulations were used to refine the structure. Finally, the best model was chosen from the values of the Modeller objective function and the DOPE assessment scores. The PyMol molecular Graphics System (http://www.pymol.org) [25] was used to visualize and analyze the generated model structure.

### Site-directed mutagenesis

The RsDTE mutants were generated using a one-step PCR method with plasmid RsDTE_pET28a wild-type as the template. The primers used for mutagenesis are summarized in Additional file 1: Table S1. After amplification, the PCR reaction mixtures were treated with *Dpn*I to completely digest the template and then transformed into *E. coli* JM109 cells. All the mutations were confirmed by DNA sequencing. Genes of RsDTE mutants were expressed and proteins were purified using the method described above for wild-type enzyme.

## Results and discussion

### Gene cloning, overexpression of RsDTE and purification of enzyme

The DTE gene was amplified by PCR using the genomic DNA of *R. sphaeroides* as a template. The results showed that this gene contained 885 bp of a complete open reading frame encoding a protein (295 amino acids, 31.75 kDa). This gene was deposited in GenBank with the Accession Number NC_007494.2. The recombinant plasmid (pET28a-RsDTE) was confirmed by double-enzyme digestion with *Nde*I and *Eco*RI (Additional file 1: Figure S1) and gene sequencing. The results show that the RsDTE gene was subcloned into pET28a plasmid, and the expression vector was constructed successfully.

**Fig. 1:**
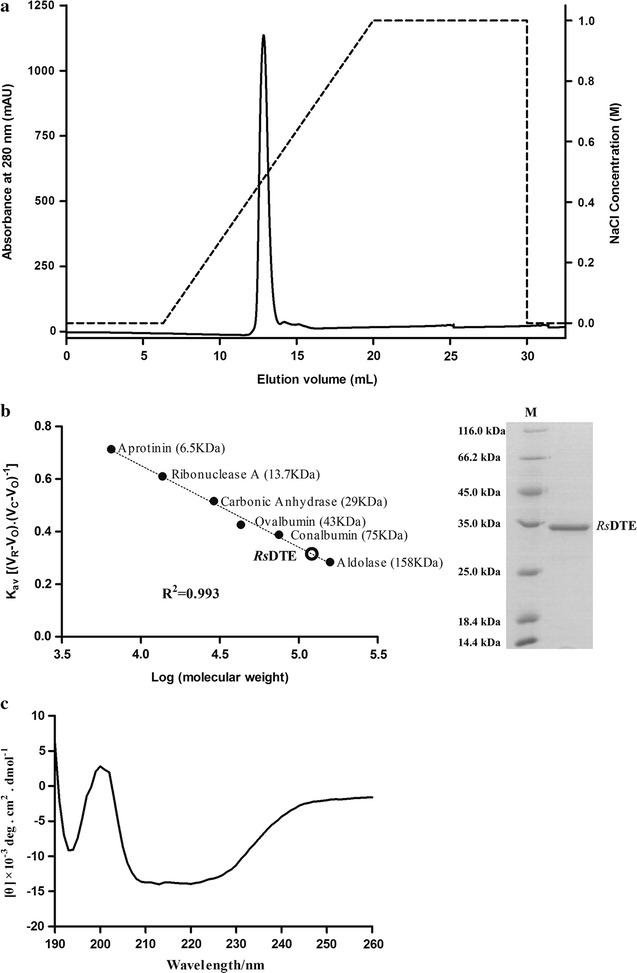
Purification of RsDTE by anion-exchange (**a**) and size-exclusion chromatography (**b**). CD spectra of RsDTE (**c**). 1 mg/mL of six standard protein markers (mass weight: 6.5, 13.7, 29, 43, 75 and 158 kDa) was used for canonical plotting of gel filtration analysis. 50 and 5 μg of purified RsDTE were used for gel filtration and SDA-PAGE analysis, respectively. 0.08 mg/mL of purified RsDTE was used for CD spectroscopy

When recombinant RsDTE was overexpressed in *E. coli* BL21(DE3), soluble and insoluble fractions were separated by centrifugation after cell lysis and analyzed using SDS-PAGE (Additional file 1: Figure S2). The eluted fractions were then further purified by anion-exchange chromatography using an AKTA system (Fig. 1a). The results showed that a single major peak was detected at 280 nm in the purified protein fraction. SDS-PAGE indicated that highly purified DTE protein was obtained. The molecular mass of RsDTE in solution was estimated by size-exclusion chromatography, and a single peak corresponding to a tetramer was observed (Fig. 1b; Additional file 1: Figure S3).

The structural features of RsDTE were evaluated using CD spectroscopy (Fig. 1c and Table 1). The CD spectrum of RsDTE showed a positive absorption peak centered around 200 nm. The α-helix and β-strand regions constituted 34.5 and 11%, of the total secondary structure, respectively.

**Table 1:** Secondary-structure contents of RsDTE determined by CD spectroscopy in the wavelength region from 190 to 260 nm

Protein	Secondary structure (%)			
	α-Helix	β-Strand	Turn	Unordered
DTE	34.5	11.0	23.1	31.3

### Characterization of RsDTE and product confirmation

**Fig. 2:**
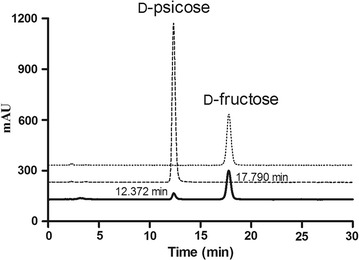
Confirmation of the enzymatic conversion product d-psicose using HPLC

The product d-psicose was confirmed by HPLC with a retention time of 12.37 min (Fig. 2). NMR spectroscopy was used to confirm identity and structure of d-psicose (Additional file 1: Figure S4). Spectral data for hydrocarbons were analyzed as follows: ^1^H NMR (400 MHz, D_2_O): δ = 3.45 (d, *J* = 11.86 Hz), 3.57–3.86 (m), 3.96–4.10 (m), 4.21 (m), 4.35 ppm (dd, *J* = 7.64, 4.68 Hz); ^13^C NMR (100 MHz, D_2_O): δ = 105.59, 103.24, 98.40, 97.66, 82.69, 82.67, 74.63, 71.70, 70.98, 70.33, 70.30, 70.21, 68.99, 65.83, 65.48, 65.05, 64.15, 63.96, 63.24, 63.04, 62.78, 62.36, 61.31, 57.95.

**Fig. 3:**
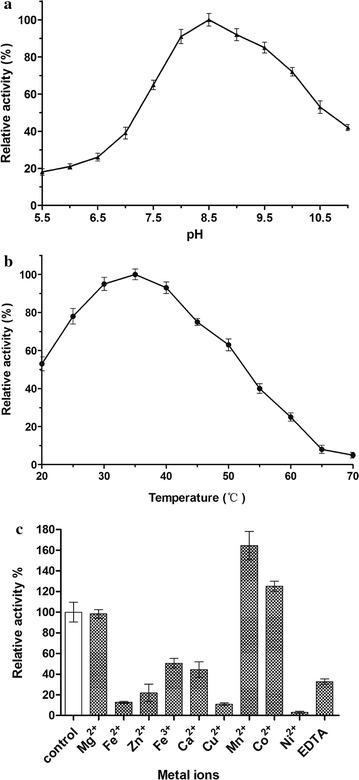
Effect of pH (**a**), temperature (**b**) and metal ions (**c**) on activity of RsDTE. The activity of purified RsDTE using AKTA system was determined in standard assay conditions as control and all of the enzyme samples were not treated with EDTA

The catalytic activity of RsDTE is highly dependent on the buffer composition, pH and temperature. The purified WT RsDTE was active at pH 5.5–11.0 and the maximal activity was observed in Tris–HCl buffer pH 8.5 (Fig. 3a). The optimal temperature for RsDTE activity was 35 °C. The activity began to decrease when the temperature was > 40 °C (Fig. 3b). The activity of RsDTE was affected by several metal ions (Fig. 3c). Adding 0.1 mM MnCl_2_ and CoCl_2_ to the reaction mixture increased the activity by 60 and 25%, respectively, which suggests that Mn^2+^ activates the catalytic reaction of RsDTE. RsDTE lost almost all activity when CuCl_2_ was added to reaction system. EDTA may not completely chelate all the metals because it still retained 33% of the residual activity. The results support the reports point that RsDTE is a metalloenzyme [13, 22].

**Fig. 4:**
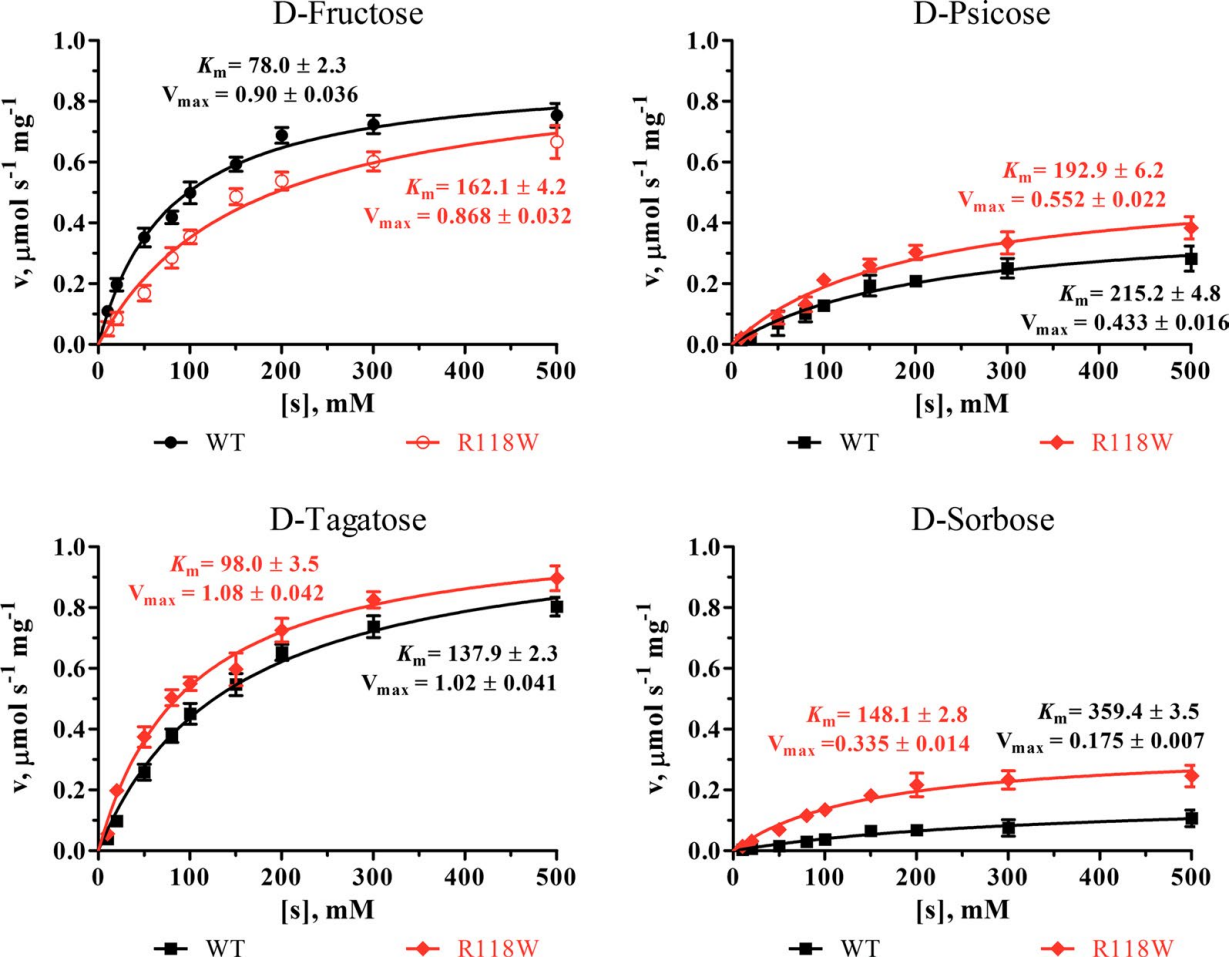
Michaelis–Menten plots of RsDTE WT and R118W mutant toward d-fructose, d-fructose, d-psicose and d-sorbose. All assays were repeated three times, and the data are shown as mean ± SD

The kinetic parameters of RsDTE on different substrates are summarized in Table 2 and Fig. 4. RsDTE was characterized by *K*_m_ values of between 78 and 359 mM, and catalytic efficiencies of between 0.02 and 0.55 s^−1^ mM^−1^. RsDTE showed a high activity toward d-fructose and a low activity toward d-sorbose.

**Table 2:** Kinetic parameters of RsDTE wild-type on four substrates

Enzymes	Substrates	*K*_m_ (mM)	*k*_cat_ (s^−1^)	*k*_cat_/*K*_m_ (s^−1^ mM^−1^)	Relative activity (%)
WT	d-Fructose	78 ± 2.3	42.60 ± 0.89	0.55 ± 0.008	100
	d-Psicose	215 ± 4.8	20.47 ± 0.63	0.10 ± 0.005	23.2 ± 1.8
	d-Tagatose	138 ± 0.8	48.31 ± 0.52	0.35 ± 0.006	69.1 ± 2.3
	d-Sorbose	359 ± 3.5	8.30 ± 0.34	0.02 ± 0.001	4.5 ± 0.4
R118W	d-Fructose	162 ± 4.2	41.05 ± 0.43	0.25 ± 0.008	58.46 ± 3.9
	d-Psicose	193 ± 6.2	26.04 ± 0.58	0.14 ± 0.008	22.78 ± 2.7
	d-Tagatose	98 ± 3.5	51.25 ± 0.54	0.52 ± 0.013	100
	d-Sorbose	148 ± 2.8	15.36 ± 0.44	0.10 ± 0.001	16.28 ± 1.6

All assays were repeated three times, and the data are shown as mean ± SD

### Crystallization of RsDTE

We obtained the apo-RsDTE crystals (Additional file 1: Figure S5) in crystallization conditions as shown in Additional file 1: Table S2 after 3 days. The crystallization conditions were further refined in different pHs and precipitants as followings: (A) 10% PEG 8000, 0.1 M HEPES pH 7.5, 8% ethylene glycol; (B) 12% PEG 20,000, 0.1 M MES pH 6.5; and (C) 12% PEG 8000, 0.1 M HEPES pH 7.5, 8% ethylene glycol at 20 °C in 96-well plates (Additional file 1: Figure S6). X-ray diffraction data were collected to about 5–6 Å resolution. Additional file 1: Figure S7 shows an X-ray diffraction image of the RsDTE crystal. The crystal needs to be further refined to obtain higher resolution for structure determination.

### Phylogenetic tree analysis of RsDTE

A search for enzymes with different amino acid sequence identities above 40% was performed using BLAST. These sequential and homologous proteins were compared with RsDTE (Additional file 1: Figure S8). RsDTE was very similar in sequence to its homologs in *Pannonibacter phragmitetus* (WP_050473007.1), *Paracoccus alcaliphilus* (SEN75178.1) and *Poseidonocella pacifica* (SFB17294.1), which shares the sequence identities of 84, 82 and 77% with these family members, respectively. The crystal structures of DTE from *P. cichorii*, *A. fabrum* str. C58, *C. cellulolyticum* H10 and *T. maritime* were determined, however, they only shared 28, 30, 36 and 20% sequence identity with RsDTE, respectively.

### Homology modeling of RsDTE

**Fig. 5:**
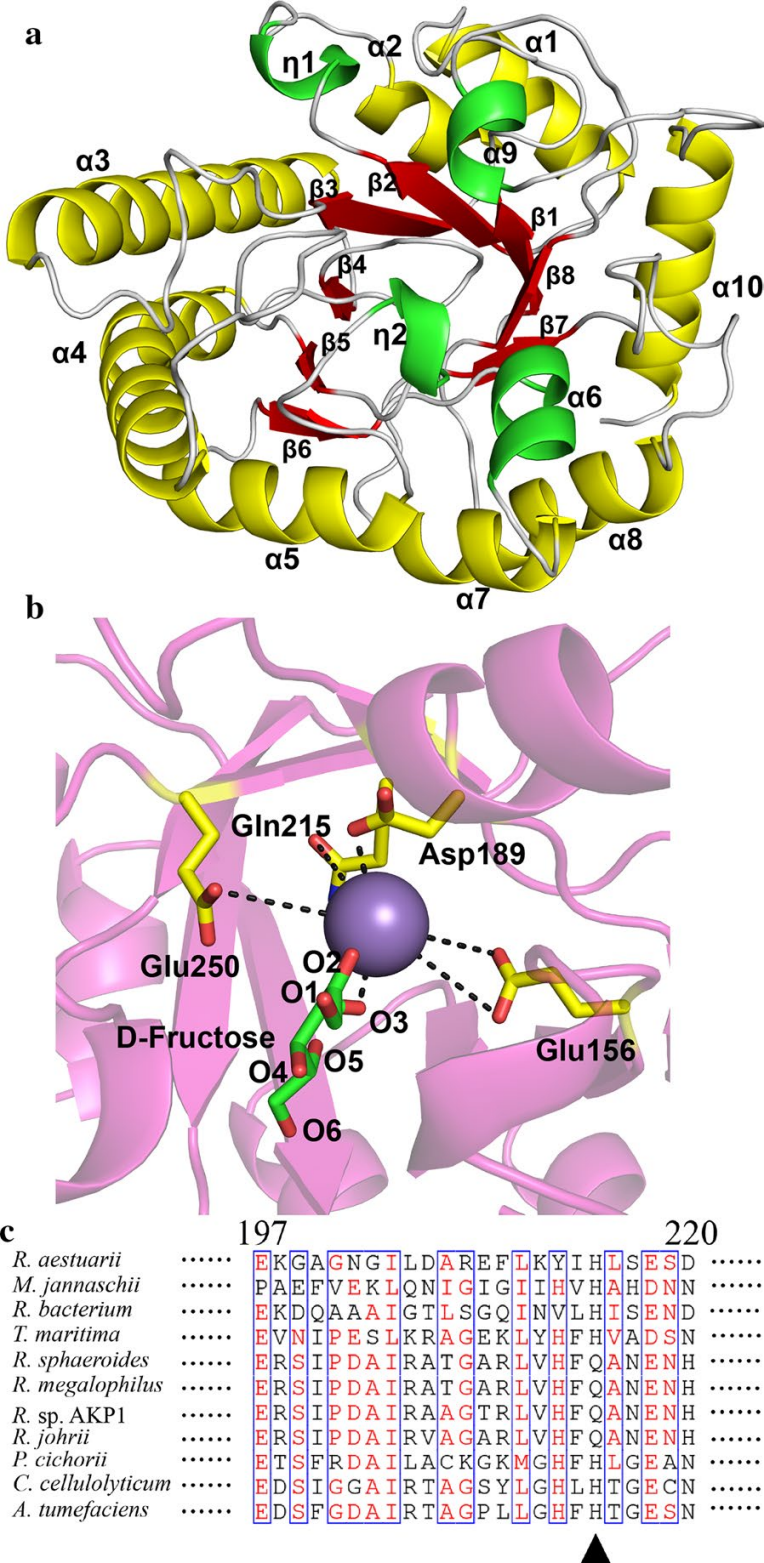
Ribbon representation of the RsDTE homology structure (**a**) and overview of the active site (**b**). The central β-strands and α-helices of TIM barrel-fold are shown in red and yellow, respectively. The additional α-helices are colored as green. The loops are colored as white. Catalytic residues are shown as yellow sticks. The substrate d-fructose is shown as green sticks. Mn(II) is displayed as a purple sphere. **c** Sequence alignment of RsDTE with from DTEs/DPEs from *Rhodobacter aestuarii*, *Methanocaldococcus jannaschii*, *Rhodobacteraceae bacterium*, *Thermotoga maritime*, *Rhodobacter sphaeroides*, *Rhodobacter megalophilus*, *Rhodobacter* sp. AKP1, *Rhodobacter johrii*, *Pseudomonas cichorii*, *Clostridium cellulolyticum*, *Agrobacterium tumefaciens*

We generated a structural homology model of RsDTE using the crystal structure of d-tagatose 3-epimerase from *P. Cichorii* (PDB ID: 2QUL, 1.79 Å), which has 31% sequence identity to the target protein RsDTE [15]. The structure of RsDTE (Fig. 5a) contained 8 β-strands, 10 α-helices and two 3_10_ helices. RsDTE possessed the characteristic TIM-barrel (β/α)_8_ fold consisting of eight repetitive units of β-strand/α-helix as the main structural motif, with a cluster of β-strands surrounded by α-helices in the center of the molecule. The other two α-helices (α6 and α9) and two 3_10_ helices were packed together along with the TIM barrel.

### Characteristics of the active site

The modeling structure of RsDTE shows that there were four residues, Glu156, Asp189, Gln215 and Glu250, around the metal-binding site, which form a hydrogen bond network for the hydride transfer reaction (Fig. 5b). This result strongly suggests that these residues constitute the catalytic tetrad of RsDTE, which is partly conserved in the DTE/DPE superfamily (Fig. 5c). Three residues of Glu156, Asp189 and Glu250 were completely conserved. However, Gln215 was not conserved, in which histidine is positioned, instead of glutamate, in *P. cichorii*, *A. tumefaciens*, *C. cellulolyticum* and *T. maritime*. The substrate d-fructose coordinated Mn(II) in a bidentate manner using its O2 and O3 groups, which form a distorted octahedral coordination geometry complex (Fig. 5b). This modeling structure supports the previously proposed mechanism of deprotonation/protonation at C3 of substrate by two Glu residues (Glu156 and Glu250) [14, 15, 18].

### Proposal interactions between enzyme and substrates

**Fig. 6:**
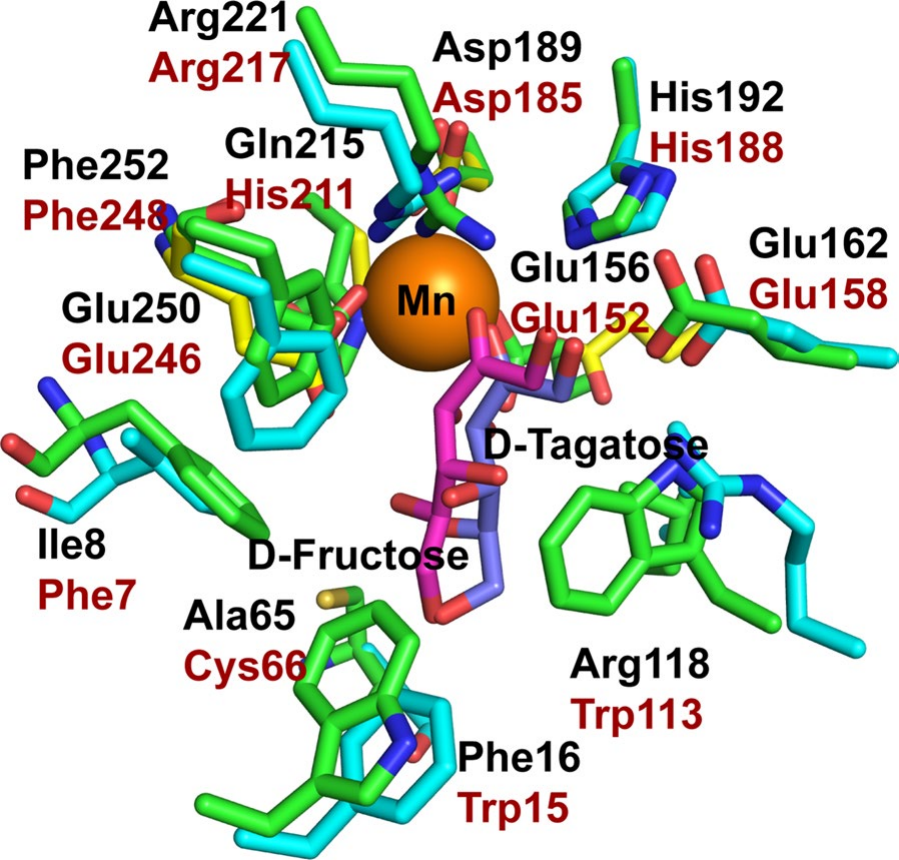
Residues at substrate binding site of RsDTE homology structure. Residues of RsDTE and *P. cichorii* DTE are shown as cyan and green sticks. The substrate d-fructose and d-tagatose are shown as magenta and purple sticks, respectively. Mn(II) is displayed as an orange sphere

The O1 of substrate d-fructose formed hydrogen bonds with His192 and Glu162 to help the correct metal coordination of the substrate (Fig. 6). The O2 formed hydrogen bonds with His192 and Arg221. Glu156 formed hydrogen bonds with O3, and Glu250 directed its OE2 atom to a hydrogen atom attached to C3. Because d-fructose has the same configurations of C1, C2 and C3 as d-tagatose, the interactions between d-fructose at the 1-, 2- and 3-positions and the enzyme were very similar to those in other DTE/DPE family enzymes [14, 15, 18]. However, the residues, which interact with d-fructose/d-tagatose at 4-, 5- and 6-positions were not conserved with these family enzymes (Fig. 6; Additional file 1: Figure S9). Ile8 (Trp in *P. cichorii*, *A. tumefaciens and* Tyr in *C. cellulolyticum*), Ala65 (Cys in *P. cichorii* and Gly in *A. tumefaciens and C. cellulolyticum*), Phe16 (Trp in *P. cichorii*, *A. tumefaciens* and *C. cellulolyticum*) and Phe252 (Phe in *P. cichorii*, *A. tumefaciens* and *C. cellulolyticum*) may form a hydrophobic pocket to interact with O4, O5 and O6 of d-fructose/d-tagatose. Arg118 (Trp in *P. cichorii*, *A. tumefaciens* and *C. cellulolyticum*) formed a unique hydrogen bond with O4, which prefers to recognize d-fructose than d-tagatose. To investigate the role of Arg118 in substrate recognition of RsDTE, we mutated Arg118 to tryptophan. RsDTE wild-type showed lower Michaelis–Menten constant (*K*_m_), lower turnover number (*k*_cat_), but higher catalytic efficiency (*k*_cat_/*K*_m_) values for d-fructose than for d-psicose. The *k*_cat_/*K*_m_ for d-fructose was 5.5-fold higher than for d-psicose, indicating that RsDTE highly catalyzed d-fructose, although it was a d-tagatose 3-epimerase. However, RsDTE_R118W mutant led to the decreased catalytic activity compared with the wild-type enzyme toward d-fructose (Table 2). The *k*_cat_/*K*_m_ for d-tagatose was about twofold higher than for d-psicose. Notably, R118 W showed 1.5-fold higher catalytic efficiency toward d-tagatose than wild-type, implying that Arg118 may regulate the substrate specificity. The unique hydrogen bond with Arg118 and O4 of d-fructose may be broken when muted to Trp. The strengthened hydrophobic interaction may attribute to the recognition of d-tagatose, d-psicose and d-sorbose (Fig. 6). The structure information supports the biochemical data that Arg118 regulates the substrate specificity.

## Conclusions

The purified d-tagatose 3-epimerase from *Rhodobacter sphaeroides* catalyzes the epimerization of d-fructose to d-psicose at the C3 position. RsDTE shows optimal conditions in Tris–HCl pH 8.5 at 35 °C. RsDTE was crystallized under refined crystallization conditions. Arg118 around the substrate-binding site was investigated, and R118 W improves the substrate recognition and activity. Consistent with biochemical data, R118 forms a hydrogen bond with O4 of d-fructose. The research of RsDTE in its active form provides a valuable tool for solving this enzyme’s crystal structure in the future.

## Additional file

**Additional file 1.** Additional tables and figures.
