# Supplementary material for: Biochemical analysis and the preliminary crystallographic characterization of d-tagatose 3-epimerase from Rhodobacter sphaeroides
Source: Microb Cell Fact. 2017 Nov 9;16:193. doi: 10.1186/s12934-017-0808-4 (PMC5679380; doi:10.1186/s12934-017-0808-4)
Supplement: Supplementary file 1 — Additional file 1. Additional tables and figures. [file 12934_2017_808_MOESM1_ESM.docx]

Additional Information for

Biochemical analysis and the preliminary crystallographic characterization of D-tagatose 3-epimerase from *Rhodobacter sphaeroides*

Zhengliang Qi^a,b,c^, Zhangliang Zhu^c^, Jian-Wen Wang^c^, Songtao Li^c^, Qianqian Guo^c^, Panpan Xu^c^, Fuping Lu^a,b,c,d*^, Hui-Min Qin^a,b,c*^

^a^Key Laboratory of Industrial Fermentation Microbiology, Ministry of Education, P. R. China;

^b^Tianjin Key Laboratory of Industrial Microbiology, P. R. China;

^c^College of Biotechnology, Tianjin University of Science and Technology, P. R. China;

^d^National Engineering Laboratory for Industrial Enzymes, Tianjin 300457, P. R. China

*Corresponding authors: F. Lu; H.-M. Qin

Tel: +86-22-60601958. Fax: +86-22-60602298

E-mail: lfp@tust.edu.cn; huiminqin@tust.edu.cn

**Table S1** Primers used for the construction of recombinant RsDTE.

| Primer name | Sequence of primer |
| --- | --- |
| R118W_F | GTCTTCGCCGGC**TGG**CCGCCCTTCCCC |
| R118W_R | GGGGAAGGGCGG**CCA**GCCGGCGAAGAC |

**Table S2** Crystallization conditions are listed as following.

| Crystal kits | Position | Crystallization conditions |
| --- | --- | --- |
| C. S. | B5 | 30% PEG 4000; 0.1 M Tris-HCl, pH 8.5; 0.2 M Lithium sulfate |
| C. S. | F10 | 12% PEG 20,000; 0.1M MES, pH 6.5 |
| C. S. | H1 | 10% PEG 8,000; 0.1M HEPES, pH 7.5; 8% Ethylene glycol |
| JCSG+ | E12 | 10% PEG 8,000; 0.1M Imidizole, pH 8.0 |
| Wizard | A1 | 20% PEG 8,000; CHES, pH 9.5 |
| Wizard | A12 | 20% PEG 1,000; 0.1M Imidazole, pH 8.0; 0.2 M Ca(OAc)_2_ |
| Wizard | D9 | 20% PEG 3,000; Acetate, pH 4.5 |
| Index | D3 | 30% v/v Jeffamine ® ED-2001, pH 7.0; 0.1 M HEPES, pH 7.0 |

C.S. means crystal screen

**
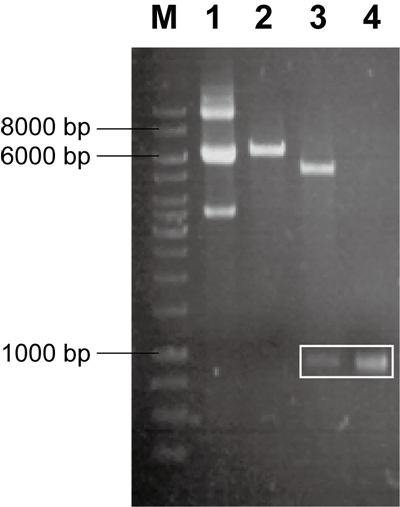
**

**Figure S1** PCR product of DTE-pET28a and enzyme digestion.

1: plasmid of DTE-pET28a extracted from JM109; 2: enzyme digestion by single restriction endonuclease; 3: enzyme digestion by double restriction endonuclease; 4: PCR product of DTE-pET28a

**
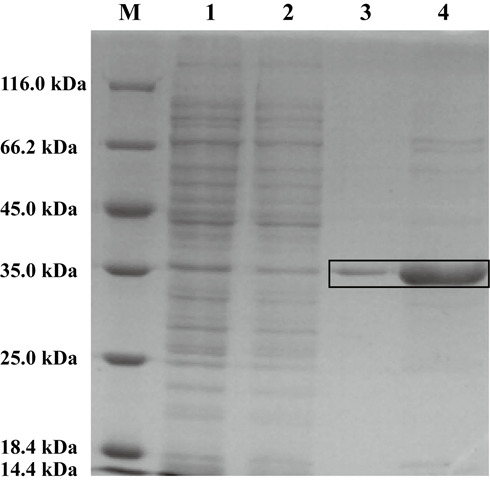
**

**Figure S2** SDS-PAGE analysis of DTE. Lane 1: supernatant; Lane 2: precipitant; Lane 3: resin trapped with DTE; Lane 4: elution.


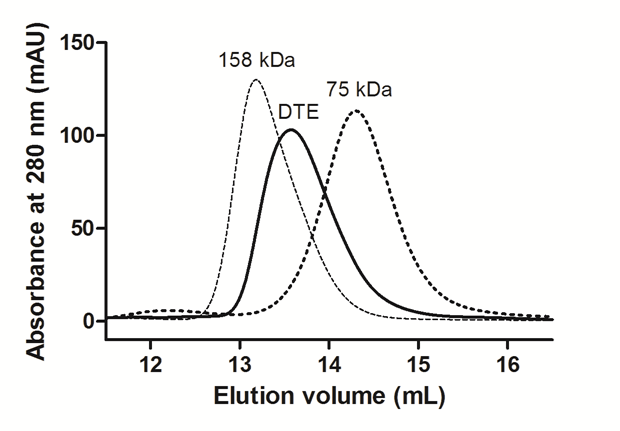


**Figure S3** Purification of RsDTE by size-exclusion chromatography.


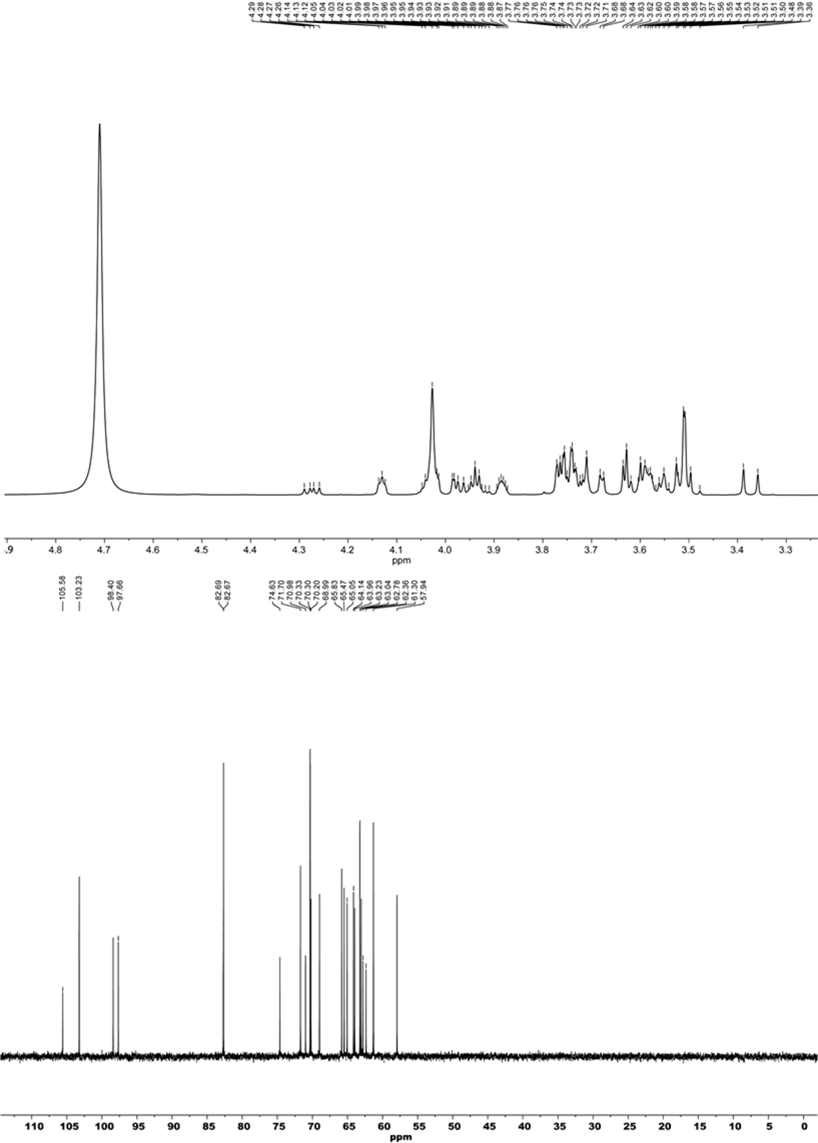


**S4** Spectral data for hydrocarbons analyzed.

**
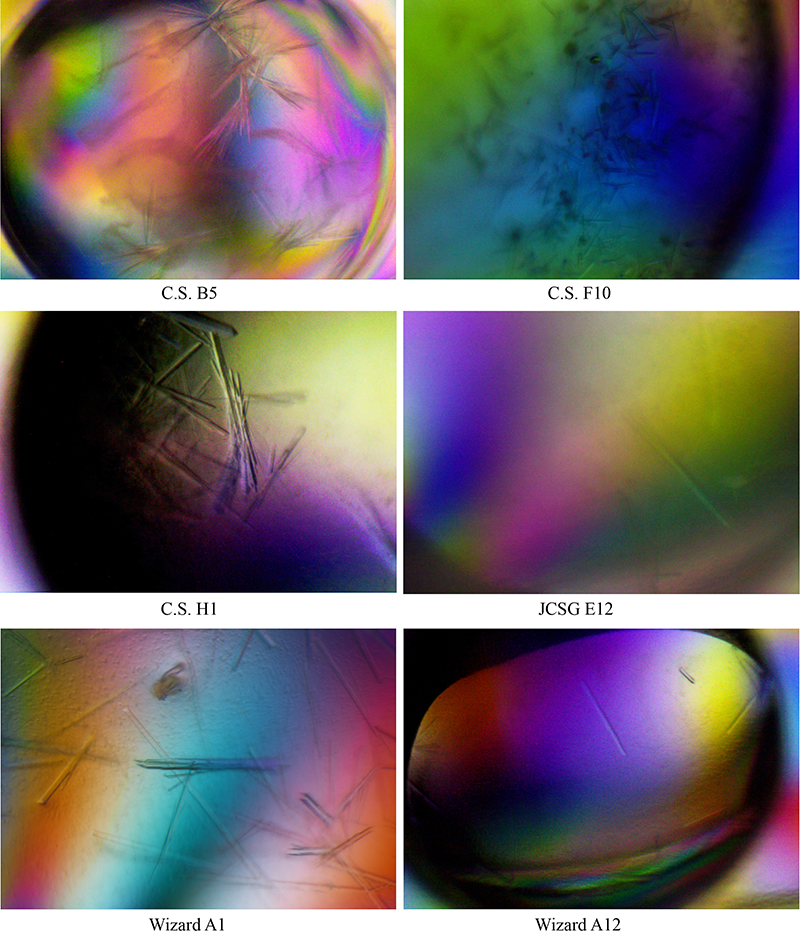
**

**Figure S5** Crystallization conditions screened with crystal kits.


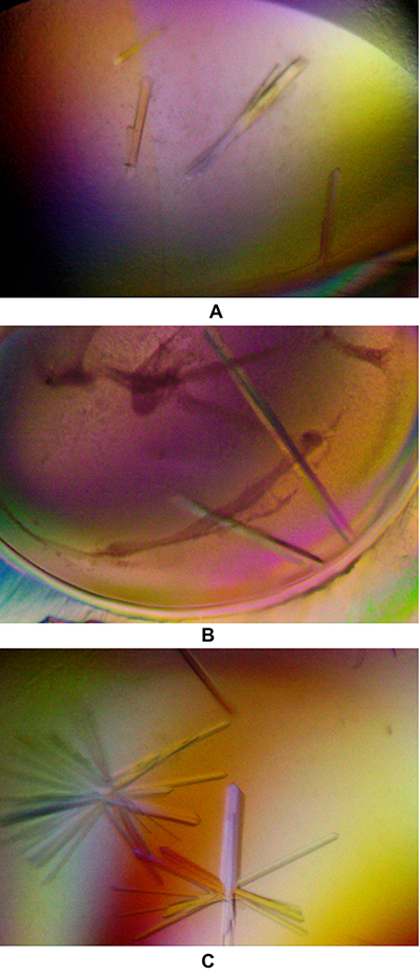


**Figure S6** Crystals of RsDTE under refined crystallization conditions.

**
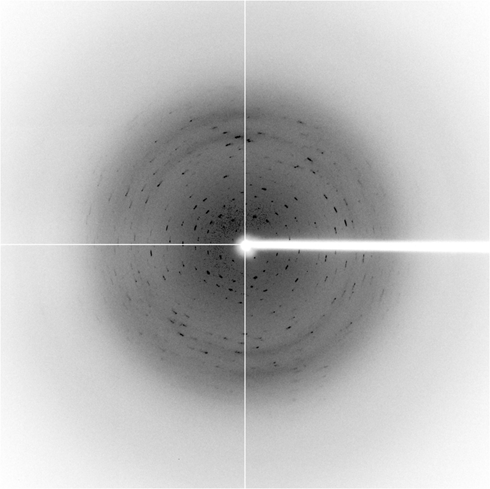
**

**Figure S7** X-ray diffraction image from RsDTE crystal.

**
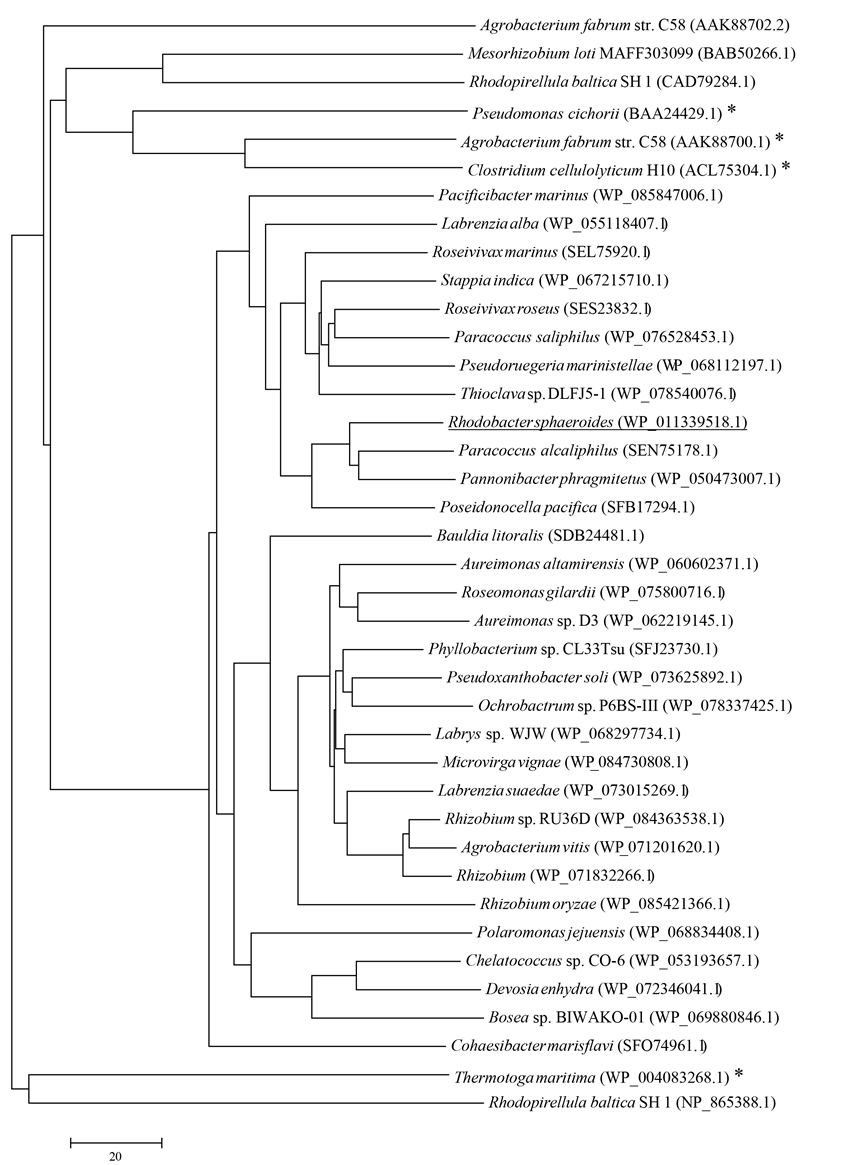
**

**Figure S8** Phylogenetic tree of RsDTE and the enzymes with amino acid sequences similar to RsDTE. The determined crystal structures of DTEs were marked as asterisk.


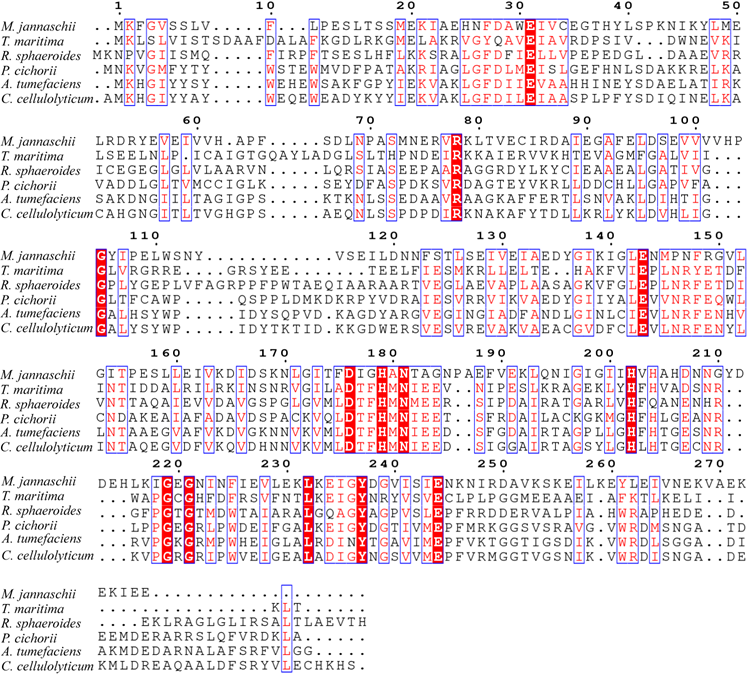


**Figure S9** Amino acid sequence alignments of D-tagatose 3-epimerase from different strains.
